# Active interaction strategy generation for human-robot collaboration based on trust

**DOI:** 10.1186/s42492-025-00198-7

**Published:** 2025-06-23

**Authors:** Yujie Guo, Pengfei Yi, Xiaopeng Wei, Dongsheng Zhou

**Affiliations:** 1 Key Laboratory of Advanced Design and Intelligent Computing (Ministry of Education), School of Software Engineering, Dalian University, Dalian, 116622 China; 2 School of Computer Science and Technology, Dalian University of Technology, Dalian, 116024 China

**Keywords:** Human-robot collaboration, Visual language model, Human-robot trust, Tree

## Abstract

In human-robot collaborative tasks, human trust in robots can reduce resistance to them, thereby increasing the success rate of task execution. However, most existing studies have focused on improving the success rate of human-robot collaboration (HRC) rather than on enhancing collaboration efficiency. To improve the overall collaboration efficiency while maintaining a high success rate, this study proposes an active interaction strategy generation for HRC based on trust. First, a trust-based optimal robot strategy generation method was proposed to generate the robot’s optimal strategy in a HRC. This method employs a tree to model the HRC process under different robot strategies and calculates the optimal strategy based on the modeling results for the robot to execute. Second, the robot’s performance was evaluated to calculate human’s trust in a robot. A robot performance evaluation method based on a visual language model was also proposed. The evaluation results were input into the trust model to compute human’s current trust. Finally, each time an object operation was completed, the robot’s performance evaluation and optimal strategy generation methods worked together to automatically generate the optimal strategy of the robot for the next step until the entire collaborative task was completed. The experimental results demonstrates that this method significantly improve collaborative efficiency, achieving a high success rate in HRC.

## Introduction

With the rapid development of robotics and artificial intelligence, human-robot collaboration (HRC) has gradually become an important research direction in industry [1, 2], medical care [3, 4], and services [5–7]. The objective of the HRC is to ensure that robots can safely and efficiently collaborate while sharing workspaces with humans. Consequently, advanced technologies, such as reinforcement learning [8–11] and machine learning [12, 13] have been widely used to significantly improve the safety, efficiency, and intelligence levels of HRC.

However, with human participation in collaborations, the role of trust in HRC has received increasing attention [14, 15]. Studies have shown that introducing trust into a HRC can improve its success rate [16, 17]. Currently, the methods of using trust to improve the success rate in HRC can be divided into two categories: one is to optimize the task allocation strategy through trust to improve the task success rate [18, 19], and the other is to guide the optimization of robot behavior strategy through trust to maximize the team’s cumulative rewards, thereby improving the task success rate [17, 20, 21]. However, most of these methods focus on how to complete the task and improve the success rate in HRC, while ignoring improvements in collaboration efficiency. In actual HRC tasks, there are two situations that reduce the efficiency of HRC: the first is unnecessary intervention, that is, humans intervene unnecessarily when robots can complete tasks independently; the second is untimely intervention, that is, when robots cannot complete tasks independently, humans do not intervene in time, resulting in task failure or repeated attempts. These situations interrupt task execution and increase the number of steps required to complete the task, thereby reducing the overall efficiency of HRC. In addition, trust computing was used to improve HRC. However, the existing trust-computing methods have limitations in practical applications. For example, a trust calculation method based on physiological data must obtain human physiological data using sensors. Although this method can directly obtain data to calculate trust values, it interferes with human comfort during the collaborative process. Most trust-based methods require the performance data of robots during collaboration as input to calculate trust values; however, the technique of obtaining such data vary across different collaborative tasks.

Therefore, this study proposes an active interaction strategy generation for HRC based on trust, which aims to use trust to enhance the predictability of human actions and reduce interruptions caused by redundant intervention and delayed decision making; thus, improving the overall efficiency of collaboration. First, a trust-based optimal robot strategy generation method was designed. This strategy was used to model the HRC under different robot strategies, and the optimal strategy of the robot was calculated based on the modeling results. In this study, we assumed that human trust was only determined by the robot’s performance [17] and selected the human-robot collaborative object transportation task on a conveyor belt as a typical industrial scenario to experimentally verify the proposed strategy. To evaluate whether the proposed strategy improves the efficiency of HRC, we introduced a clear metric for collaboration efficiency. Although the task completion time is commonly used, individual differences in operational speed may introduce a bias. Therefore, instead of using absolute time, we quantified the collaboration efficiency using the number of steps required to complete the task collaboratively. This step-based metric provides a stable and objective assessment of the fluidity and efficiency of a collaborative process. Second, to calculate human’s trust, the robot’s performance must be obtained. Additionally, the rapid development of vision language model (VLM) technology has demonstrated powerful capabilities in visual perception and natural language processing, providing a new technical approach for the collection and analysis of robot performance data. Therefore, for the task scenario in this study, we combined visual language model technology and designed a robot performance evaluation method based on a visual language model to support trust calculations.

Based on this thesis, we aim to develop a systematic approach to trust perception and robot behavior optimization within HRC. Our objective is to promote seamless HRC and provide new theoretical and practical support for more efficient and intelligent collaboration.

The main contributions of this study are as follows:

- The optimal robot strategy generation method based on trust is proposed, which includes two parts: trust-based modeling of the HRC process and the optimal robot strategy calculation method. This method aims to employ the tree to model HRC process under different robot strategies, and calculate the optimal strategy of the robot’s next step based on all the modeling results. Every time the robot completes the operation of an object, this method generates the optimal strategy for the robot’s next step, until the entire collaborative task is completed.
- The VLM-based robot performance evaluation method is proposed, which includes subscene reasoning for HRC based on VLM and robot’s performance evaluation based on the sequence of HRC subscenes. This method can quantify the performance of the robot, and the quantification results can be input into the trust model to calculate the human’s current trust.

**Method for evaluating human-robot trust**

Collaboration efficiency is an important issue in the field of HRC, and trust is an important factor that affects collaboration efficiency [22]. To cooperate efficiently and seamlessly with humans, robots must be able to identify human trust [23]. Presently, trust generation methods are divided into two categories: subjective and objective. Subjective measurement directly obtains individuals’ perceptions and evaluations of trust through self-assessment methods, such as questionnaires, whereas objective measurement relies on the analysis and evaluation of physiological signals or calculates trust values based on existing trust models.

Subjective measurement methods have many applications. For example, Anzabi and Umemuro [24] obtained the trust values of humans in four listening behaviors of robots using questionnaires to explore the impact of different listening behaviors on trust. Esterwood and Robert [25] obtained human’s trust in robots through questionnaires, and further studied whether trust repair strategies can fully restore trust. However, subjective measurement methods have limitations in dynamic collaborative environments, because frequent questionnaires or self-assessments can interrupt collaboration and affect the naturalness and efficiency of real-time interactions.

Therefore, many studies have focused on objective trust measurement methods that evaluate trust values in real-time by analyzing physiological signals or using trust models to minimize interference with collaboration. For example, Zhang et al. [26] evaluated drivers’ trust in self-driving cars using electroencephalogram (EEG) signals, whereas Hopko and Mehta [27] found that the prefrontal cortex area of the brain and its specific neural connection patterns can be used as a sign to identify whether humans trust robots. Recently, Loizaga et al. [28] comprehensively analyzed a variety of physiological signals, including EEG, galvanic skin response, respiration, and pupillometry, to explore the dynamic interaction and evolution of trust and then studied the correlation between these signals in depth. These studies provide more accurate quantitative method of trust perception through physiological data. Simultaneously, another group of researchers obtained data that affect human trust in HRC through questionnaires and used them for trust value evaluation. For example, Xu and Dudek [29] first designed a trust model that established Bayesian beliefs about human trust at each moment based on a robot’s task performance over a period, thereby calculating human trust in the robot. To comprehensively describe human-robot trust, Ahmad et al. [30] constructed a mathematical model that integrates four objective measurement factors to reflect the degree of trust in human interactions with robots from multiple dimensions. Rabby et al. [31] further integrated the performance of humans and robots to quantify trust by developing a novel time-driven trust mathematical model that considers the performance of HRC.

In summary, objective measurements based on physiological signals can provide more accurate trust assessments; however, wearing sensors in practical applications may affect the comfort of collaboration. However, objective measurement methods that calculate trust values using trust models can solve this problem. Therefore, this study adopted a method for calculating trust using trust models.

**Optimization approach for HRC based on trust**

The fundamental purpose of studying trust is to combine it with human-robot collaborative tasks such that robots can perceive the trust level of humans and dynamically adjust their own behaviors based on trust, thereby improving collaboration efficiency while ensuring safety. Currently, several studies have explored methods to optimize HRC using trust. Introducing trust into HRC can help further improve its quality and safety [14, 32, 33]. Existing methods for optimizing HRC using trust can be divided into two categories: optimizing the task allocation strategy through trust to improve the success rate in HRC, and guiding the optimization of the robot’s behavior strategy through trust to maximize the team’s cumulative rewards, thereby improving the success rate in HRC.

The objective of the first method is to leverage the respective advantages of humans and robots to complete collaboration more efficiently. For example, Ali et al. [34] first introduced trust into the design of a task allocation strategy for HRC and then further proposed a new trust-based task allocation method for heterogeneous human-robot teams in ref. [18], which can dynamically adjust the allocation of existing and new tasks over time. Considering that few studies have explored the impact of trust on multihuman-multirobot collaboration in the context of task allocation, Obi et al. [19] explored the key role of trust in task allocation in multihuman-multirobot collaboration, providing a new perspective for further optimizing task allocation strategies.

The main goal of the second method is to optimize the robot’s behavior strategy through trust to maximize the overall reward of the team. This type of research is typically based on the reinforcement learning framework. For example, Chen et al. [17] incorporated trust into a partially observable Markov decision process (POMDP) framework and calculated a robot strategy that can maximize a team’s task performance by influencing human intervention behavior. Based on the POMDP framework, Sheng et al. [20] proposed a trust-based route planning method for autonomous vehicles that modeled human-vehicle interaction as a POMDP and determined the optimal route for the autonomous vehicle by solving the optimal strategy in the POMDP. In addition, Abouelyazid [35] designed a framework that integrates multiple reinforcement learning techniques and applied it to the field of robotics to improve the safety of operations and enhance human trust in robots. Yu et al. [21] combined trust with POMDP for robot motion planning, enabling it to collaborate with humans to complete tasks and obtain an optimal strategy while satisfying specific time-sequence constraints.

In summary, current trust-based HRC optimization methods are primarily focused on improving the success rate of tasks but often ignore the key factors that affect the efficiency of HRC. Specifically, most existing studies have failed to fully consider the impact of human intervention (especially unnecessary or untimely) on the collaborative process. For example, unnecessary intervention refers to human intervention even when the robot can complete a task independently, and untimely intervention refers to the failure of human intervention when the robot encounters difficulties. Both of these situations interrupt the task execution process and increase the time required to complete the task, thereby reducing the overall collaboration efficiency. Therefore, it is necessary to develop trust-based methods to address these issues.

## Methods

**Fig. 1:**
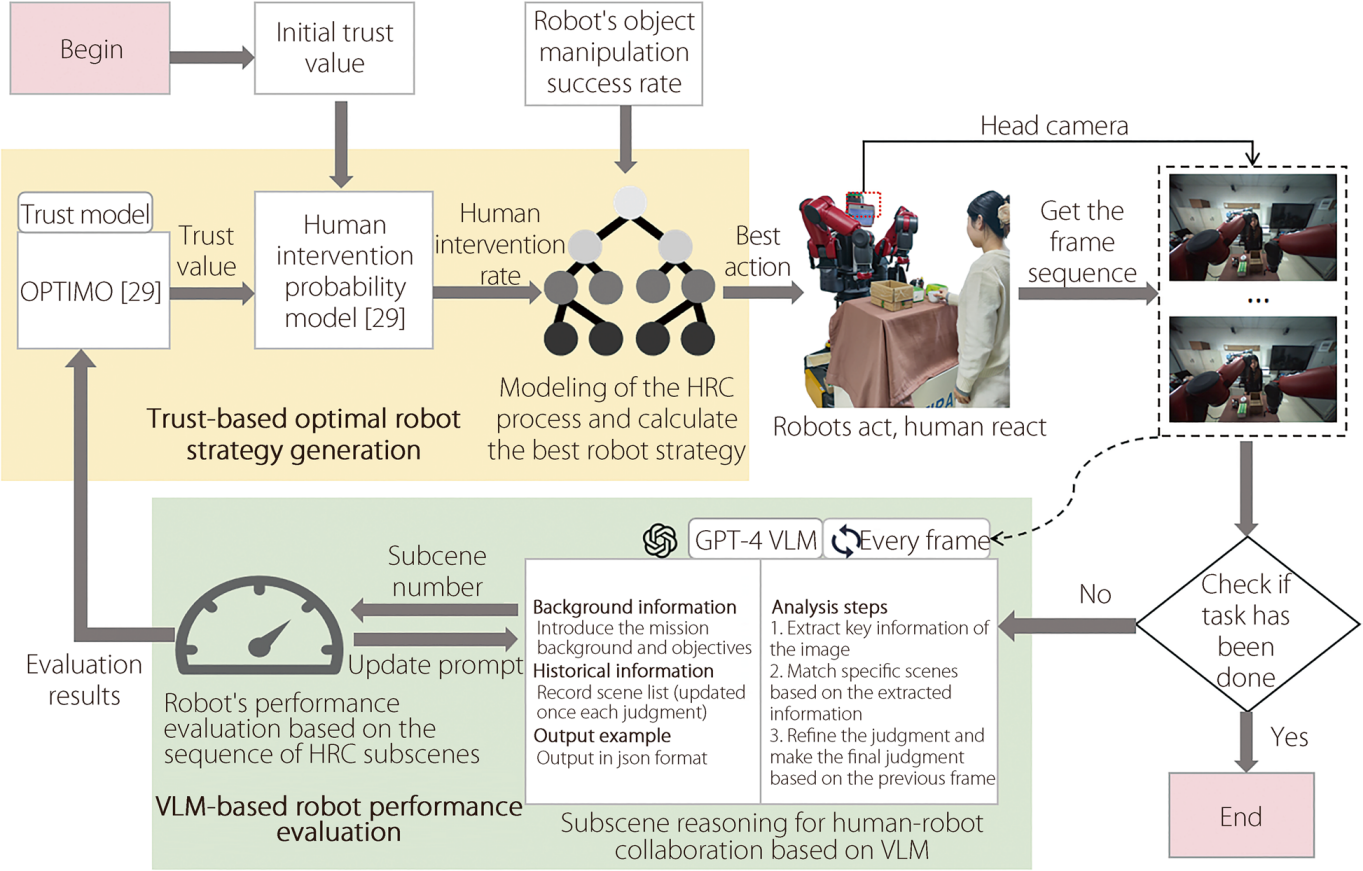
Framework diagram of active interaction strategy generation for HRC based on trust

This paper proposes an active interaction strategy generation for HRC based on trust, as shown in Fig. 1. The framework consists of two parts: a trust-based optimal robot strategy generation method and a VLM-based robot performance evaluation method. These are used to dynamically calculate the optimal strategy of the robot in collaboration and capture its performance.

### Trust-based optimal robot strategy generation method

This subsection introduces in detail the structure and function of the trust-based optimal robot strategy generation method, which includes two parts: trust-based modeling of the HRC process and optimal robot strategy calculation. The objective of this method is to employ a tree to model the HRC process under different robot strategies, and then calculate the optimal strategy of the robot’s next step based on these modeling results. By constructing the tree, the possible HRC processes under each robot strategy are clearly presented, thus providing a basis for evaluating and selecting the optimal strategy for the robot’s next step.

#### Trust-based modeling of the HRC process

In human-robot collaborative tasks, the robot’s actions are typically predefined. For example, in the task of human-robot collaborative cleaning of a desktop, the robot’s actions are mainly grabbing and placing, and its strategy is composed of different combinations of these actions. However, it is difficult to directly determine the best robot strategy because the choice of the optimal strategy depends on the execution effect of the action itself as well as on the combined influence of multiple factors, such as the behavior of the collaborative object and the complexity of the task goal. Therefore, to determine the optimal strategy, these factors must be considered comprehensively during the modeling process.

This study adopts a tree-based strategy-generation method. This is because the Markov Decision Process must solve the optimal value function to obtain the optimal solution, and the value function is inherently nonlinear. In general, obtaining a closed-form solution is infeasible, requiring approximation through an iterative algorithm. Therefore, guaranteeing an accurate optimal solution is difficult. However, our method is exhaustive. Tasks with a small state space can exhaust all the possible strategies within an acceptable time cost to obtain an optimal solution to the problem.

In actual HRC, humans may intervene in the robot’s operation, and the robot may fail when operating the object. Therefore, when constructing a tree, the probabilities of human intervention and robot failure must be considered. The probability of human intervention reflects the level of human trust in the robot’s current performance. If the rate of intervention is high, humans may not trust the robot’s performance sufficiently and thus, more likely to intervene. To describe the relationship between human trust and intervention behavior, this study references a human intervention probability prediction model [29]. Specifically, the intervention probability is formulated using a logistic conditional probability distribution that considers a human’s current trust value and the change in trust value. These factors were combined linearly with the learned weights and passed through a sigmoid function to produce a normalized intervention probability. This model effectively captures the dynamic relationship between variations in trust and human intervention behavior. Human trust was calculated using the trust model [29] based on the robot performance evaluation result p (the calculation process of p is described in detail in “VLM-based robot performance evaluation method” subsection). Second, the success rate of a robot manipulating objects depends on the characteristics of the object and the performance of the robot. Consequently, the success rate was calculated based on the robot and object used in the experiment.

By combining the two key factors of human intervention rate and success rate of the robot manipulating objects, we formulated the construction rules of the tree. Considering that this study focuses on the typical industrial scenario of human-robot collaborative object transportation tasks on the conveyor belt, the following rules are formulated based on this scenario and can be applied to the same type of HRC tasks.

1. When the robot performs the ‘grasp’ action: Human can choose to intervene or not, and the analysis is conducted according to the probability of human intervention.
  If human intervenes, because the robot and human try to grasp the object simultaneously, to avoid potential safety risks, the robot will stop when it observes the human performing the grasping operation, thereby ensuring the successful completion of the task. In this case, human intervention may occur in two situations: one is that the robot could have successfully grasped the target object, and the other is that the robot would have failed to grasp the target object. Therefore, in the tree, this situation is subdivided into two branches: “Could have been grasped successfully” and “Would have failed” to finely analyze the impact of human intervention on the grasping task.
  If human does not intervene, at this time only the robot performs the grasping operation, and the task may succeed or fail. The specific result depends on the success rate of robot manipulating the current object, and further analysis is performed accordingly.
2. When the robot performs the ‘non-grasp’ action: Similarly, human can choose to intervene or not.
  If human intervenes, at this time, because the robot chooses not to grasp, only the human performs the grasping action, and the task is finally successfully completed.
  If human does not intervene immediately, because the scenario we are targeting is HRC in industrial scenarios, these tasks have clear goal orientation and completion time limits. In this type of collaboration, when the robot chooses not to grasp or is unable to perform task owing to a malfunction, human will assume the responsibility and take over to ensure task completion.

**Fig. 2:**
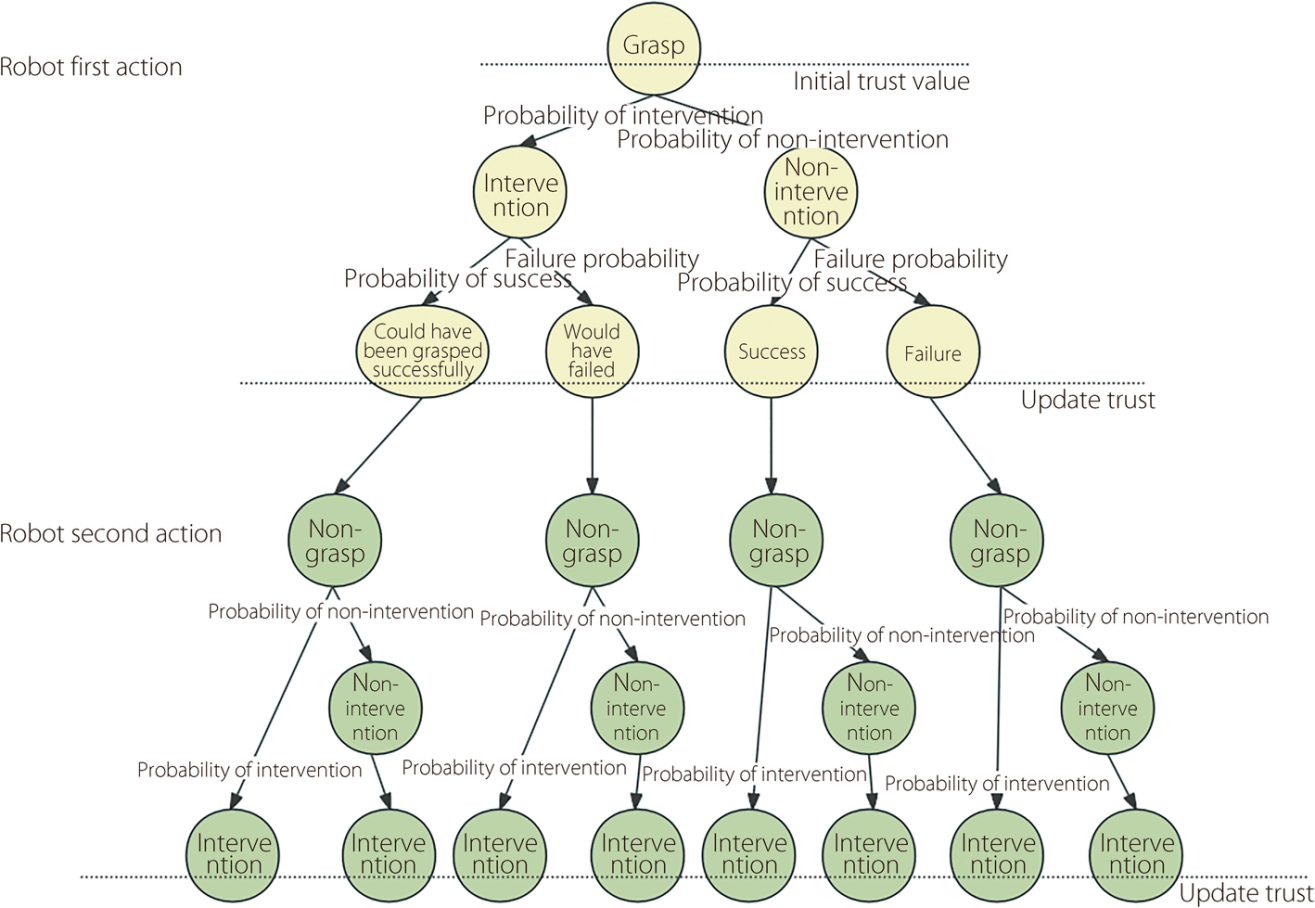
Example diagram of tree’s structure

The following Fig. 2 shows an examples of tree built according to the above rules. The figure shows a tree with a depth of 2, where it is assumed that the first action of a robot strategy is ‘grasp’ and the second action is ‘non-grasp.’

#### The optimal robot strategy calculation

In the previous subsection, we constructed a corresponding tree for each robot strategy to simulate the process of robots collaborating with humans using different strategies. The main task described in this subsection is to evaluate these trees and select an optimal robot strategy. In an actual HRC, unnecessary or untimely human intervention can significantly increase the number of steps required to complete a task, thereby reducing the overall collaboration efficiency. Therefore, we introduced a time evaluation metric to quantify and compare the effectiveness of the different strategies. Specifically, we used the number of steps required to complete the task to quantify the task completion time.

It is assumed that the number of steps required to complete the task in each collaboration situation is as shown in Table 1, which quantifies the number of steps spent in different collaboration situations and analyzes the reasons for spending these steps in each collaboration situation. This table provides a basis for calculating the number of steps required to complete a task in the experiment in “Experimental design” subsection.

**Table 1:** Quantification and analysis of the number of steps spent on tasks under different collaboration situations

Collaboration situation	Further situation	Step spent	Reason
Robot grasps and human don’t intervene	Grasping success	1	Normal time consumption
	Grasping failure	3	No timely intervention
Robot grasps and human intervene	Could have been grasped successfully	2	Unnecessary intervention
	Would have failed	1	Normal time consumption
Robot doesn’t grasp	Human don’t intervene	3	No timely intervention
	Human intervene	1	Normal time consumption

In Table 1, the number of steps in each case is set as follows: the robot autonomously grasps and successfully completes the task, which requires only one step; if the robot fails to grasp autonomously and the humans do not intervene, the robot must reposition, grasp, and place again, which requires at least three steps. In Table 1, we set it to three steps; if the robot can grasp successfully on its own but humans intervene, the intervention forces the robot to stop and wait for the human to act, resulting in an additional two steps; on the contrary, if the robot may fail to grasp, but humans intervene promptly to complete the task, the human intervention directly corrects the grasping process, requiring only one additional step (equivalent to “human one-time completion”). When the robot does not grasp and the human does not intervene, because neither party assume action, a waiting step and a human reaction step will be generated, and the task will eventually be completed after the human reacts. The entire process requires three steps: when the robot does not grasp, the human directly intervenes and completes the task; that is, the human completes it in one step, which requires one step. It should be noted that although the operational complexity of each step may vary, this study focuses on whether there are behaviors that affect the efficiency of collaboration, such as untimely or unnecessary human intervention, rather than the specific execution difficulty of each step. Therefore, we use the number of steps as an abstract indicator to measure the efficiency of collaboration, aiming to reflect the smoothness and effectiveness of the collaboration process. Based on this goal, ignoring the complexity differences between steps will not affect the effectiveness of the indicator in evaluating the overall collaboration efficiency.

The tree (as shown in Fig. 2), each leaf node corresponds to a specific collaboration situation under the robot strategy. Combined with the number of steps cost of each cooperation situation listed in Table 1, we calculate the tree corresponding to each strategy and evaluate the average number of steps cost of each strategy through a mathematical expectation. The formula is as follows:

1 $$\begin{aligned} E\left( X\right) = \sum \limits _{k=1}^{N}{X}_{k}{P}_{k} \end{aligned}$$

where ${X}_{k}$ represents the number of steps spent on each path in the tree, ${P}_{k}$ is the probability of occurrence of path *k* and N represents the number of leaf nodes under a particular strategy.

This method compares the expected values of all the robot strategies and selects the strategy with the smallest expected value as the optimal strategy. If there are multiple strategies with equal expected values, one is randomly selected and the first action in the strategy is used as the optimal action for the robot’s next step. This study defines the process of human and robot collaborating to complete the operation of a single object as a “time step.” After each time step, human’s trust is re-evaluated, and the optimal strategy is recalculated to output the optimal strategy for the robot’s next step.

### VLM-based robot performance evaluation method

This subsection introduces in detail the structure and function of the robot’s performance evaluation method based on VLM, which includes two parts: subscene reasoning for HRC based on VLM and robot performance evaluation based on the sequence of HRC subscenes, which are responsible for inferring the collaborative subscene in each picture and judging the robot performance. This method aims to assess the robot’s performance by reasoning about key subscenes during the task execution process and analyzing the fixed sequence of these subscenes.

#### Subscene reasoning for HRC based on VLM

This subsection introduces subscene reasoning for HRC based on VLM. The VLM used in this method is ChatGPT 4, which can process text and image information for efficient reasoning. For the human-robot collaborative subscene ${S}_{t}$ at each moment, the model must perform comprehensive reasoning based on the observed image ${O}_{t}$, prompt ${R}_{t}$ and historical information ${H}_{t-1}$. The reasoning process is defined as follows:

2 $$\begin{aligned} {S}_{t}=VLM({O}_{t}, {R}_{t}, {H}_{t-1}) \end{aligned}$$

**Collaborative subscene** ${S}_{t}$: The ${S}_{t}$ refers to the collaborative subscene number outputted by the VLM at the current moment. Each number corresponds to a specific collaborative subscene. The tasks in this study are divided into seven subscenes, as listed in Table 2. The order of subscenes in which the robot successfully completed the task was 1–5–6, while the order of subscenes in which it failed was 1–5–7–8, 1–5–7, and 1–5-1 (i.e., the object was picked up and then failed again). The order of subscenes in which humans successfully completed the task was 1–2–3–4.

**Table 2:** Seven subscenes of the human-robot collaborative object transportation task on the conveyor belt

Number	Subscene	Human_action	Robot_action	Object_position
1	Robot is about to grasp	1	False	False
2	Human intervention	2	False	False
3	Human is grasping	3	False	False
4	Object is successfully grasped by human	2	False	True
5	Robot is grasping	1	True	False
6	Object is successfully picked by robot	1	False	True
7	Object falls	1	False	False

**Observed image** ${O}_{t}$: It is composed of the image captured by the head camera of the baxter robot at the current time *t*. The image was stored through the subscription function of the Robot Operating System (ROS) image storage node as a direct input for model reasoning.

**Prompt word** ${R}_{t}$: The prompt adopts the chain of thought prompting [36] method, which guides the model to complete the inference of complex tasks in stages through phased and step-by-step reasoning. Figure 1 shows the design example of ${R}_{t}$, which includes the following three reasoning stages:

1. Information extraction. This stage mainly obtains three types of information from the image ${O}_{t}$:
  “Human_action”: extract human’s action to determine whether human have hand intervention during the collaboration and whether they hold objects in their hand. If no human’s hand is detected, recorded as ‘1’; if human’s hand is detected but does not hold an object, recorded as ‘2’; if human’s hand appears and holds an object, recorded as ‘3.’
  “Robot_action”: extract robot’s action to determine whether the robot is gripping objects. If the robot’s gripper successfully grasps the object and has left the desktop, recorded as ‘true’; if the robot’s gripper does not grasp the object, or has grasped the object but remains on the desktop, recorded as ‘false.’
  “Object_position”: extract the position of the object and determine whether the object has been correctly placed in the target area. If the object to be grasped is within the target area, recorded as ‘true’; if the object to be grasped is outside the target area, recorded as ‘false.’
2. Preliminary subscene determination. This stage classifies the current image ${O}_{t}$ into some collaborative subscenes based on the information extracted in the previous stage and the definition of the collaborative subscenes ${S}_{t}$.
3. Subscene disambiguation and finalization. When there is ambiguity in subscene determination, historical information ${H}_{t-1}$ can be combined to eliminate uncertainty and finally accurately determine the current collaborative subscene. Specifically, when the three types of information corresponding to the image ${O}_{t}$, namely Human_action, Robot_action and Object_position, match multiple subscenes, it is necessary to combine the historical information ${H}_{t-1}$ to eliminate ambiguity. For example, in Table 2, the two types of information of the 1st and 7th subscenes are the same, thus it is impossible to determine which of the two subscenes it belongs to based on the information of the current image alone. Because there is a causal relationship between each subscenes, the order in which the subscenes occur is fixed. For example, if no sequence number is recorded in the historical information ${H}_{t-1}$, the subscene in which the current image is located is determined to be 1. As the subscene 7 (object falls) cannot appear alone, and its prerequisite is that the object has been grasped. If subscene sequence number 5 (robot is grasping) appears in ${H}_{t-1}$, the subscene in which the current image is located is determined to be 7.

**Historical information** ${H}_{t-1}$: refers to time series data recorded in the list, which stores the sequence of collaboration subscene numbers from the start of the task to the current moment. It is expressed as ${H}_{t-1}=[{s}_{1},{s}_{2},\ldots ,{s_{t-1}}]$, where *s* indicates a specific collaboration subscene number.

#### Robot performance evaluation based on the sequence of HRC subscenes

In this subsection, the functions and evaluation processes of the proposed method are introduced in detail. The method has two core functions: one is to dynamically update historical information promptly and the other is to evaluate and output the robot’s performance. For the specific reference algorithm, refer to Algorithm 1.

Specifically, every time the VLM completes the analysis of the image frame, it receives the analysis result ${S}_{t}$ output by the VLM and adds it to the historical information list ${H}_{t-1}$ of the prompt in real time, thereby generating complete historical information ${H}_{t}$ at the current moment. This mechanism effectively enhances the contextual understanding ability of the VLM in multiframe image sequences, ensuring that it can infer the current task state based on the order of the previous and next frames.

In addition, this method evaluates and outputs the robot’s performance by comprehensively analyzing the subscene sequences of the current and previous frames. Specifically, each time ${S}_{t}$ is received, this method evaluates the task status of the robot based on historical information ${H}_{t}$. Consider the experimental task in this study as an example, the robot must collaborate with the human operator to place objects on the conveyor at the specified location. According to the sequence of subscene numbers in which the robot successfully completes the task, the human completes the task, and the robot fails, as described above, this matches the sequence recorded in ${H}_{t}$ with these three defined sequences to evaluate whether the robot succeeds or fails in the task. After each evaluation, ${H}_{t}$ is reset to prepare for the assessment of the next object.

The final output of this method is the performance of the robot $p\in \left\{ 1,0\right\}$, where 0 indicates task failure and 1 indicates task success. The output is used as the input of the OPTIMO trust model [29].

**Figure:**
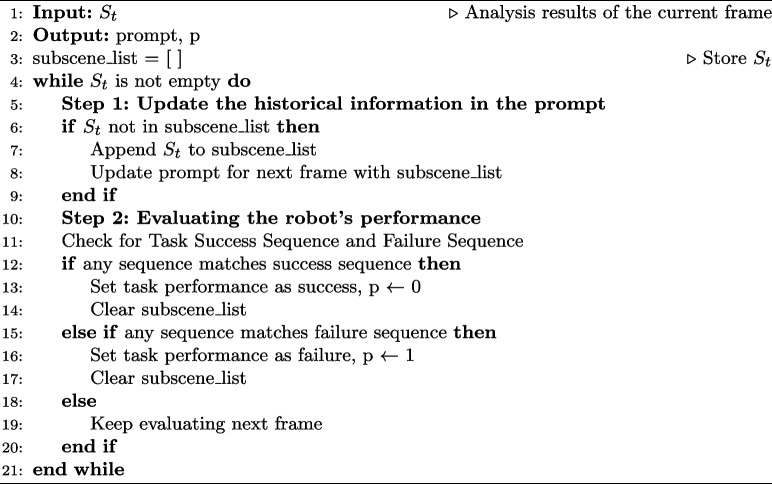
**Algorithm 1** Robot’s performance evaluation based on the sequence of HRC subsceness

### Experimental settings

**Fig. 3:**
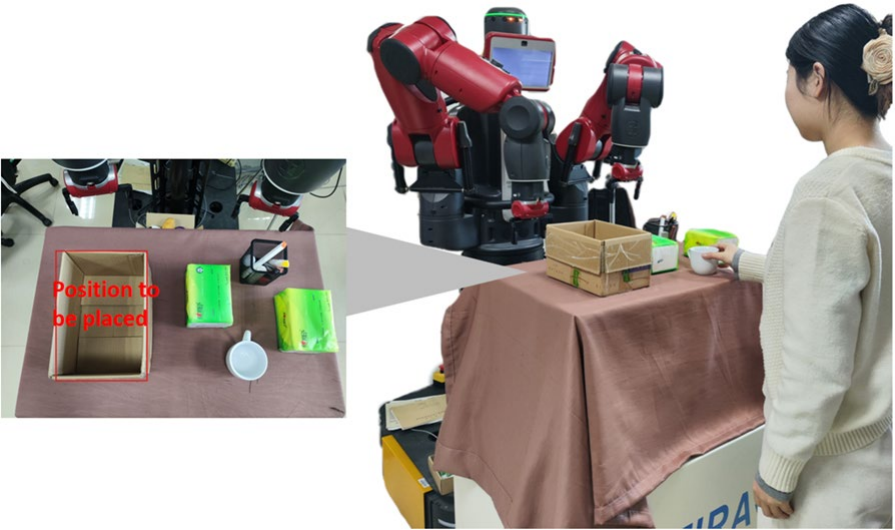
Experimental setup

To verify the efficiency of the trust-based robot active interaction strategy, we deployed it on a baxter robot and conducted the related experiments. As shown in Fig. 3, this study uses the baxter robot as the experimental platform, where the red, green, blue (RGB) camera mounted on the head captures the HRC scene, and the RGB camera on the arm acquires positional information of the desktop objects to support precise grasping actions. The resolution of both images was 1280 $\times$ 800 pixels. A laptop equipped with an NVIDIA GeForce 4060 graphics processor was used for image processing and robot control. The control program was written in Python, while communication and control were achieved using the ROS. The experimental workbench was located within the effective working range of the Baxter, and an experimenter was present in the workspace to participate in the collaborative tasks.

### Experimental design

In this study, we designed a simple experimental environment to simulate the scenario of a human-robot collaborative object transportation task on a conveyor belt. Specifically, the experiment simulated an assembly line task by having the robot and human operator collaborate in sequence to grasp objects on a table. This task requires the robot to collaborate with humans to place objects conveyed on the conveyor belt at a specified location. In this experiment, three different types of objects were selected as operation objects: paper tissue, a glass cup, and an iron-pen holder. The grasping success rates of the objects on the baxter robot were 100%, 35%, and 85%, respectively. Typically, in HRCs, these success rates are known to the robot; however, the human operator does not require memorization. Therefore, the experiments were conducted under these conditions.

In addition, to evaluate the effectiveness of the proposed trust-based active interaction strategy in improving the efficiency of HRC, we conducted a comparative experiment under two conditions: one adopting an active interaction strategy based on trust (experimental group, EG) and the other adopting a random strategy (control group, CG). Consider that there are currently no other research methods available for direct comparison with our strategy proposed in this paper, we designed a “random strategy” as the CG in our experiments to verify the effectiveness of the proposed method in enhancing HRC efficiency. Specifically, the “random strategy” refers to the robot randomly selecting between the actions of ‘grasp’ and ‘non-grasp’ at each task step, without considering the human’s trust value, thereby forming the random strategy. To ensure human safety during the experiment, if the robot detects any human intervention, it immediately stops the current operation to prevent potential harm to humans. As the “random strategy” lacks a clear decision-making logic, its performance is entirely dependent on randomness. Therefore, under the condition that all experimental settings are identical, if our method significantly outperforms the “random strategy,” it can strongly demonstrate that this advantage stems from the effectiveness and intelligence of the proposed strategy itself.

Consequently, we further set up three experimental scenarios, controlling for different influencing factors (such as initial trust value and object operation order, etc.), to compare the collaboration efficiency of the control and EGs under various conditions. The specific experimental settings were as follows:

**Experiment 1**: Verify the impact of random factors in the collaboration on the experimental results. Under the same initial trust value, even if repeated experiments are conducted, the human intervention time and number of interventions may vary owing to the unpredictability of the collaboration process. This experiment was conducted to isolate these random factors and ensure the robustness of the experimental conclusions. Therefore, 15 groups of comparative experiments were designed to cover all possible situations: when the number of interventions was one, intervention was conducted at four different time steps, and corresponding CGs were set up, for a total of four groups of experiments; when the number of interventions was two, any combination of two time steps was selected for intervention, and a CG was set up, for a total of six groups of experiments; when the number of interventions was three, any combination of three time steps was selected for intervention, and a CG was set up, for a total of four groups of experiments; when the number of interventions was four, intervention was conducted at all-time steps, and a CG was set up, for a total of one group of experiments. The experiments were replicated ten times per group, and the average was calculated for further analysis. By comparing the results of the control and EGs, we evaluated whether the changes in the time and number of interventions affected the experimental results to verify whether the experimental results were affected by random factors.

**Experiment 2**: Verify the efficiency of the strategy and explore the impact of the initial trust value on the experimental results. This experiment aimed to verify the efficiency of the trust-based robot active interaction strategy and analyze the impact of the initial trust value on the experimental results. Because the EG adopts this strategy and the initial setting of the trust value directly affects the execution of the strategy, the use of a single trust value for experiments may not fully prove the universality of the experimental results, and may affect its reliability. To understand the potential impact of the initial trust value on the experimental results, this experiment selected 10 discrete values (from 0.1 to 1, increasing by 0.1 every time) in the trust value interval [0,1] for the experiments and set up a CG. The CG adopts a random strategy instead of a trust-based strategy. Finally, the experimental results of these 10 groups were compared and analyzed with those of the CG.

**Experiment 3**: Verify the impact of different operation orders of objects on the experimental results and verify the effectiveness of the strategy. Because the first two experiments were conducted under a fixed order of objects, this experiment used an unfixed order of objects to eliminate the impact of the order of object operation on the experimental results. Ten groups of experiments were conducted, in which the order of objects in each group was randomly generated, and the experimental and CGs were compared with the same order of objects. The EG adopted a trust-based active interaction strategy, whereas the CG adopted a random strategy. The experimental process and evaluation indicators were consistent with those of the first two experiments, which assumed that the initial trust value for human was 0.7. These settings were determined based on the results of the second experiment, which examined the performance of the proposed strategy under different initial trust values. The results show that when the trust value is 0.7, the HRC achieves the highest efficiency. Therefore, in the third experiment, we adopted 0.7 as the initial trust value to further investigate how different object operation sequences affect task outcomes.

We first eliminated the possibility that the experimental results were affected by random experimental conditions through Experiment 1 and then further designed Experiment 2 to verify the impact of different initial trust values on the experimental results and prove the effectiveness of the strategy. Finally, Experiment 3 verified the effect of different item orders on the experimental results. These three experiments verify the effectiveness of the proposed strategy.

## Results and Discussion

Before presenting the experimental results, it should be clarified that although the success rate is not explicitly presented as a separate quantitative indicator in the Results section, the experimental design itself ensures that all target objects can be successfully grasped in each trial. Specifically, even if the robot fails to grasp an object for the first time, the system triggers a second grasp or allows timely human intervention to ensure the completion of each task. Based on this setting, the focus of our analysis is to evaluate whether the proposed trust-based active interaction strategy can improve the efficiency of HRC under this premise. The comparative results for collaboration efficiency under different experimental conditions are shown below. The results of Experiment 1 are shown in Tables 3, 4, 5; the results of Experiment 2 are shown in Table 6; and the results of Experiment 3 are shown in Table 7.

Table 3 shows a comparison of the number of steps required to complete the task between the experimental and CGs at different intervention time steps when the number of interventions was one. Considering the average figures in the table, it is clear that all the EGs showed significant advantages over the CG. First, when the intervention was performed in the first time step, the average number of steps required to complete the task in the EG was reduced by approximately 26.83% compared with the CG. Among the 10 experiments, the EG completed tasks in a notably shorter time compared to the CG in 9 experiments, and only in the 7th experiment did the CG assume a slightly shorter number of steps compared to the EG. This occurrence can be accounted for by the principles outlined in the method referenced in “Trust-based optimal robot strategy generation method” subsection: the optimal strategy calculated by our method aims to maximize the probability of completing the task with the least steps, rather than simply pursuing the absolute shortest number of steps to complete the task. Therefore, the strategy selected in this study is a trade-off between fewer steps required to complete the task and a higher probability of success. Therefore, in individual experiments, the number of steps required to complete the task in the EG may not be the least; however, overall, the strategy of the EG was significantly better than that of the CG in terms of performance. When the human operator intervened at the second time step, the average number of steps required to complete the task in the EG was reduced by approximately 39.73% compared to the CG, which was more significant than the results of the comparative experiment that intervened at the first time step. When the human operator intervene in the third time step, the average number of steps required to complete the task in the EG was reduced by approximately 26.67% compared to the CG. In 10 experiments, the number of steps required to complete the task in the EG was significantly lower than that in the CG. When the human operator intervene in the fourth time step, the average number of steps required to complete the task in the EG was reduced by approximately 27.27% compared to the CG.

**Table 3:** Four groups of comparative experiments with intervention

Experiment number	Intervention at time step 1		Intervention at time step 2		Intervention at time step 3		Intervention at time step 4	
	Number of step (CG)	Number of step (EG)	Number of step (CG)	Number of step (EG)	Number of step (CG)	Number of step (EG)	Number of step (CG)	Number of step (EG)
1	9	**7**	7	**5**	11	**7**	10	**5**
2	8	**4**	10	**4**	9	**5**	11	**7**
3	8	**4**	10	**4**	11	**7**	8	**7**
4	7	**6**	8	**4**	8	**7**	11	**7**
5	11	**7**	6	**4**	8	**7**	9	**7**
6	10	**7**	8	**7**	10	**7**	9	**7**
7	**4**	5	6	**4**	8	**7**	**7**	**7**
8	8	**7**	8	**4**	8	**7**	**5**	**5**
9	9	**5**	**4**	**4**	11	**7**	11	**7**
10	8	**7**	6	**4**	8	**5**	7	**5**
Average number of step	8.2	**5.9**	7.3	**4.4**	9.2	**6.6**	8.8	**6.4**

Table 4 compares the number of steps required to complete the task for the experimental and CGs at distinct intervention time steps when the interventions were applied twice. From the average values in the table, all EGs showed significant advantages over the CG, suggesting that under the same number of interventions, this strategy can effectively reduce the number of steps required to complete the task and optimize the collaboration effect. When humans intervene in the first and second time steps, the optimization effect is the most significant. In this group of experiments, the minimum average number of steps required to complete the task in the EG reached 4.7, and the average number of steps required to complete the task in the EG was reduced by approximately 27.7%. Because the operation success rate of the corresponding items in the second time step is low, if the human operator intervenes, it can effectively avoid the waste of steps caused by the failure to intervene in time; thus, the number of steps required to complete the task in this EG is the shortest.

**Table 4:** Six groups of comparative experiments with 2 intervention times

Experiment number	Intervention at time step 1 and 2		Intervention at time step 1 and 3		Intervention at time step 1 and 4		Intervention at time step 2 and 3		Intervention at time step 2 and 4		Intervention at time step 3 and 4	
	Number of step (CG)	Number of step (EG)	Number of step (CG)	Number of step (EG)	Number of step (CG)	Number of step (EG)	Number of step (CG)	Number of step (EG)	Number of step (CG)	Number of step (EG)	Number of step (CG)	Number of step (EG)
1	7	**4**	7	**6**	10	**7**	8	**5**	5	**4**	9	**4**
2	6	**4**	9	**7**	6	**5**	8	**6**	8	**5**	10	**5**
3	5	**4**	8	**6**	8	**5**	8	**6**	8	**5**	9	**5**
4	9	**6**	9	**6**	9	**6**	9	**6**	9	**6**	9	**5**
5	8	**6**	8	**6**	8	**5**	8	**5**	7	**4**	6	**4**
6	5	**4**	5	**4**	5	**4**	6	**4**	9	**7**	8	**5**
7	6	**4**	6	**4**	9	**6**	7	**5**	7	**5**	9	**5**
8	7	**4**	7	**4**	10	**6**	6	**4**	6	**4**	6	**5**
9	6	**6**	9	**7**	6	**4**	5	**4**	5	**4**	10	**6**
10	6	**5**	6	**4**	9	**7**	4	**4**	7	**6**	9	**5**
Average number of step	6.5	**4.7**	7.4	**5.4**	8.0	**5.5**	6.9	**4.9**	7.1	**5.0**	8.5	**4.9**

..

Table 5 illustrates the differences in the number of steps required to complete the task between the experimental and CGs at different intervention time-steps when the number of interventions was three or four. From the average values in the table, it can be observed that all the EGs showed significant advantages over the CG, which further verifies the efficiency of this strategy in improving task efficiency. Compared with Tables 3 and 4, when the number of interventions increased to three or four, the average number of steps required to complete a task in the EG decreased overall. However, this downward trend was not positive, because an increase in the number of interventions introduced unnecessary interventions. In addition, because of our experimental design, when the number of interventions reached three or more, it was inevitable that interventions would be made on items with high success rates and no need for intervention; thus, the number of steps required to complete the task could not be continuously optimized.

**Table 5:** Five groups of comparative experiments with 3 and 4 intervention times

Experiment number	Intervention at time step 1, 2, and 3		Intervention at time step 1, 2, and 4		Intervention at time step 1, 3, and 4		Intervention at time step 2, 3, and 4		Intervention at time step 1, 2, 3, and 4	
	Number of step (CG)	Number of step (EG)	Number of step (CG)	Number of step (EG)	Number of step (CG)	Number of step (EG)	Number of step (CG)	Number of step (EG)	Number of step (CG)	Number of step (EG)
1	5	**4**	8	**6**	8	**5**	6	**5**	6	**5**
2	7	**5**	**4**	**4**	7	**5**	6	**5**	5	**4**
3	6	**4**	6	**4**	9	**6**	6	**5**	7	**5**
4	7	**5**	7	**5**	7	**5**	7	**6**	5	**4**
5	6	**4**	6	**4**	6	**4**	5	**4**	**4**	**4**
6	6	**5**	6	**4**	6	**4**	7	**6**	7	**6**
7	**4**	**4**	7	**4**	7	**5**	8	**7**	5	**4**
8	5	**4**	8	**6**	8	**5**	**4**	**4**	6	**4**
9	7	**6**	**4**	**4**	7	**5**	6	**5**	5	**4**
10	**4**	**4**	7	**6**	7	**5**	5	**4**	5	**4**
Average number of step	5.7	**4.5**	6.3	**4.7**	7.2	**4.9**	6.0	**5.1**	5.5	**4.4**

From the experimental data in the three tables mentioned earlier, the findings of the 13 EGs demonstrated that the EG achieved better results than the CG. This result verifies that the efficiency of the experimental results is based on the proposed strategy rather than the accidental effect caused by specific experimental conditions (such as intervention time or number of times) and excludes the possibility that the experimental results are affected by random experimental conditions.

**Table 6:** Average number of steps required to complete the task for the EG and the CG under different trust values

Group	Trust value	Experiment amount	Average number of step
CG		10	8.3
EG (ours)	0.1	10	**4.9**
	0.2	10	**5.5**
	0.3	10	**5.7**
	0.4	10	**6.5**
	0.5	10	**6.6**
	0.6	10	**6.3**
	0.7	10	**5.9**
	0.8	10	**6.5**
	0.9	10	**6.7**
	1.0	10	**6.8**

Table 6 presents the average number of steps required to complete a task for both the EGs and CGs under different trust conditions. The results demonstrate that the number of steps required to complete the task of the EG was significantly lower than that of the CG, which indicates that the strategy adopted by the EG performed well in improving the efficiency of our strategy. Note that when the trust value is low (such as 0.1–0.3, etc.), although the average number of steps required to complete a task is small, it is accompanied by an abnormally high human intervention rate. This is not true HRC but is owing to the extreme distrust of humans in robots and frequent task takeover. In this case, the robot has almost no room for autonomous operation and the collaborative relationship is weakened. Therefore, it is not sufficient to rely on the average task completion time to measure the collaborative effect; it is necessary to combine the human intervention rate for comprehensive consideration. From a comprehensive analysis, when the trust value was 0.7, the average number of steps required to complete the task was 6.0. Excluding the low-trust groups, the HRC effect of this group is the most significant. This shows that the trust value should not be too high or too low, but should be within a reasonable range to effectively improve the efficiency of HRC. This conclusion provides data support for the regulation of trust value in HRC as well as provides important inspiration for the design of trust optimization strategies in practical applications.

Table 7 lists the effects of different object manipulation sequences on the experimental results. The findings indicate that, regardless of the manipulation order, the number of steps required to complete the task for the EG was consistently lower than that for the CG. This demonstrates the robustness of the proposed strategy in enhancing HRC efficiency, which is unaffected by variations in the object-handling order, thereby further substantiating its effectiveness.

**Table 7:** Comparison of average number of steps required to complete a task between the EGs and CGs under varying operational sequences

Object operation order	Experiment amount	Group	Average number of step
Tissue-tissue-pen holder-cup	10	EG	**5.5**
		CG	7.9
Tissue-tissue-cup-pen holder	10	EG	**5.7**
		CG	7.6
Tissue-pen holder-tissue-cup	10	EG	**6.2**
		CG	8.3
Tissue-pen holder-cup-tissue	10	EG	**6.5**
		CG	8.2
Tissue-cup-tissue-pen holder	10	EG	**6.8**
		CG	8.5
Tissue-cup-pen holder-tissue	10	EG	**6.3**
		CG	8.7
Pen holder-tissue-tissue-cup	10	EG	**5.9**
		CG	7.8
Pen holder-tissue-cup-tissue	10	EG	**6.1**
		CG	8.1
Pen holder-cup-tissue-tissue	10	EG	**6.2**
		CG	8.1
Cup-tissue-tissue-pen holder	10	EG	**5.8**
		CG	7.9
Cup-tissue-pen holder-tissue	10	EG	**6.1**
		CG	8.0
Cup-tissue-pen hodel-tissue	10	EG	**6.6**
		CG	8.4

In summary, the first experiment shows that the effectiveness of the experimental results stems from the inherent advantages of the proposed strategy rather than the accidental effects of specific experimental conditions (such as the intervention time or number of times), thus eliminating the possibility that the experimental results are affected by random experimental conditions. Therefore, the second experiment further verified that the performance of the proposed strategy was always better than that of the CG at different initial trust levels; thus, strongly proving its significant advantages in improving the efficiency of HRCs. Based on the previous two experiments, the third experiment further confirmed that the performance of the strategy was not affected by the sequence of object operations and was always better than that of the CG; thus, highlighting its stability and superiority in diverse scenarios.

This method has achieved remarkable results in improving the efficiency of HRC and enhancing the collaborative experience of human operators; however, it has certain limitations. When calculating the optimal strategy for HRC, the success rate of the robot manipulating the object is based on subjective observation and calculation of the human operator’s task performance, which lacks intelligence. In future research, we will focus on optimizing this link by introducing a more intelligent evaluation mechanism and reducing the reliance on manual observations to further improve the applicability and robustness of the method.

## Conclusions

This thesis proposes an active interaction strategy generation for HRC based on trust. It presents a systematic trust perception and robot behavior optimization framework within HRC. This strategy completes tasks efficiently as well as significantly improves the overall efficiency of HRC. This improvement optimizes the collaboration process as well as significantly enhances the experience of human collaborators, making the interaction feel more seamless and reliable. In addition, the efficiency and adaptability of this method make it highly applicable across various fields, such as intelligent manufacturing, medical health, and home services. It is expected to promote the implementation and promotion of HRC technology across various scenarios, demonstrating its broad application potential.

## Data Availability

Not applicable.

## Abbreviations

HRC: Human-robot collaboration
VLM: Vision language model
EEG: Electroencephalogram
POMDP: Partially observable Markov decision process
RGB: Red, green, blue
ROS: Robot Operating System
EG: Experimental group
CG: Control group
